# Associations of Serum Uric Acid Levels With Macrovascular and Renal Microvascular Dysfunction Among Individuals From Sub-Saharan Africa

**DOI:** 10.1001/jamanetworkopen.2021.28985

**Published:** 2021-10-14

**Authors:** Charles F. Hayfron-Benjamin, Bert-Jan van den Born, Albert G. B. Amoah, Anke H. Maitland-van der Zee, Karlijn A. C. Meeks, Erik J. A. J. Beune, Kerstin Klipstein-Grobusch, Charles Agyemang

**Affiliations:** 1 Department of Public Health, Amsterdam University Medical Centre (UMC), University of Amsterdam, Amsterdam Public Health Research Institute, Amsterdam, the Netherlands; 2 Department of Internal and Vascular Medicine, Amsterdam UMC, University of Amsterdam, Cardiovascular Sciences, Amsterdam, the Netherlands; 3 Department of Respiratory Medicine, Amsterdam UMC, University of Amsterdam, Amsterdam, the Netherlands; 4 Department of Physiology, University of Ghana Medical School, Accra, Ghana; 5 Center for Research on Genomics and Global Health, National Human Genome Research Institute, National Institutes of Health, Bethesda, Maryland; 6 Department of Medicine and Therapeutics, University of Ghana Medical School, Ghana; 7 Department of Anaesthesia, Korle Bu Teaching Hospital, Accra, Ghana; 8 Julius Global Health, Julius Center for Health Sciences and Primary Care, University Medical Center Utrecht, Utrecht University, Utrecht, the Netherlands; 9 Division of Epidemiology and Biostatistics, School of Public Health, Faculty of Health Sciences, University of the Witwatersrand, Johannesburg, South Africa

## Abstract

**Importance:**

Serum uric acid (SUA) level is associated with vascular dysfunction in Eurasian populations, but little is known about this association in individuals from sub-Saharan Africa, who have a high prevalence of both relatively high SUA levels and vascular dysfunction.

**Objectives:**

To assess the associations of SUA levels with macrovascular and kidney microvascular dysfunction in individuals of sub-Saharan African ancestry and evaluate potential factors that could mediate these associations.

**Design, Setting, and Participants:**

Cross-sectional analyses of baseline data from the multicenter Research on Obesity and Diabetes Among African Migrants study, conducted from 2012 to 2015, were performed from January to March 2021. The population included Ghanaian individuals living in Ghana and Europe.

**Exposure:**

Abnormal SUA levels.

**Main Outcomes and Measures:**

Logistic regression was used to examine the associations of SUA level quartiles with microvascular (albuminuria) and macrovascular (peripheral artery disease and coronary artery disease) dysfunction, with adjustments for age, sex, estimated glomerular filtration rate, site of residence, socioeconomic status, alcohol, smoking, diabetes, hypertension, waist-hip ratio, and total cholesterol level. Mediation analysis was performed to assess whether the association was via elevated blood pressure, hemoglobin A_1c_, and high-sensitivity C-reactive protein levels or via weight-hip ratio. The research questions were formulated after data collection.

**Results:**

A total of 4919 Ghanaian individuals (3047 [61.9%] women) aged 25-75 years (mean [SD], 46.26 [11.08] years) were included. There was a significant positive association between SUA quartiles and albuminuria, but not coronary artery disease or peripheral artery disease, after adjustment for covariates. After full adjustment, individuals in the fourth SUA quartile had higher odds of albuminuria (adjusted odds ratio [aOR], 1.54; 95% CI, 1.07-2.21), but not peripheral artery disease (aOR, 1.35; 95% CI, 0.87-2.08) or coronary artery disease (aOR, 1.09; 95% CI, 0.77-1.55), compared with individuals in the first quartile. After full adjustment, systolic and diastolic blood pressure significantly mediated the association between SUA concentrations and albuminuria, accounting for 19.4% of the total association for systolic and 17.2% for diastolic blood pressure; hemoglobin A_1c_, high-sensitivity C-reactive protein, and waist-hip ratio did not mediate this association.

**Conclusions and Relevance:**

In this cross-sectional study among a sub-Saharan African population, elevated SUA levels were significantly associated with kidney microvascular dysfunction and mediated partly through elevated blood pressure. These findings suggest that individuals from sub-Saharan Africa with elevated SUA levels may benefit from periodic screening for kidney microvascular dysfunction to aid early detection or treatment.

## Key Points

**Question:**

Are high or low serum uric acid levels associated with vascular dysfunction in individuals with sub-Saharan African ancestry?

**Findings:**

In this cross-sectional analysis that included 4919 people from Ghana, elevated serum uric acid level was significantly associated with higher odds of kidney microvascular dysfunction, but not coronary artery disease or peripheral artery disease, after adjusting for a wide range of cardiometabolic risk factors. Elevated blood pressure significantly mediated the association between high serum uric acid level and kidney microvascular dysfunction; poor glycemic control, obesity, and inflammation did not significantly mediate the association.

**Meaning:**

The findings of this study suggest that individuals from sub-Saharan Africa with elevated serum uric acid levels may benefit from periodic screening for kidney microvascular dysfunction to aid early detection or treatment.

## Teaser

This cross-sectional study examines the association between serum uric acid levels and vascular dysfunction among individuals of sub-Saharan African ancestry.

## Introduction

In the general population, atherosclerotic macrovascular diseases are common, such as coronary artery disease (CAD), peripheral artery disease (PAD), and cerebrovascular disease, and frequently complicate acute coronary syndromes, critical limb ischemia, and cerebrovascular accidents.^1,2^ Likewise, microvascular disease, including nephropathy, is prevalent and remains a leading cause of kidney failure.^3^ These vascular complications are responsible for most cardiovascular disease (CVD)–related morbidity and deaths.^1,2,3^

Data have shown that the conventional CVD risk factors are unable to fully explain the development and/or progression of vascular dysfunction.^4^ Experimental, clinical, and epidemiologic data suggest that individuals with elevated serum uric acid (SUA) levels are at increased risk of CVD and kidney dysfunction.^5,6,7,8^ Most research exploring the role of SUA levels in the pathogenesis of microvascular and macrovascular dysfunction has typically excluded sub-Saharan African (SSA) ancestry populations.^5,6^ Like most other vascular disease risk factors,^9^ the role of SUA as a potential risk factor may be associated with race and ethnicity. Relatively high rates of microvascular and macrovascular dysfunction among SSA individuals have been reported recently, which were not sufficiently explained by conventional CVD risk factors.^10,11,12,13^ In this same cohort, hyperuricemia was found to be associated with an increased 10-year CVD risk^14^; however, the association between SUA concentration and vascular dysfunction in SSA populations remains unknown. In addition, studies in other populations have typically focused on the association between high SUA concentration and vascular disease.^5,6^ A prospective study conducted in Rotterdam, the Netherlands, reported a U-shaped association between SUA levels and both all-cause and CVD mortality, suggesting that both low and high SUA concentrations may be detrimental to cardiovascular function.^15^ The mechanistic basis is partly due to the dual role of SUA as an antioxidant (intracellular) and a pro-oxidant (extracellular) to oxidative stress, depending on its localization.^16^ There also are limited data on the biological basis of the association between SUA levels and vascular dysfunction. Based on previous reports from experimental and clinical studies, hypertension,^6,7^ inflammation,^17^ obesity,^18^ and hyperglycemia^19^ could be factors that associate SUA levels with vascular dysfunction. However, epidemiologic data testing these potential mediations are limited. Using a representative sample of Ghanaian individuals, this study assessed the associations between SUA concentrations and macrovascular and microvascular dysfunction in SSA populations. Furthermore, we evaluated the mediating roles of hypertension, hyperglycemia, inflammation, and obesity in the association between SUA levels and macrovascular and microvascular dysfunction.

## Methods

### Study Design

This cross-sectional analysis, performed from January to March 2021, was based on baseline data from the multicenter Research on Obesity and Diabetes Among African Migrants study, conducted from 2012 to 2015. The research questions were formulated after data collection. The rationale, conceptual framework, design, and methods of the study have been described in detail elsewhere.^20^ In brief, the Research on Obesity and Diabetes Among African Migrants study comprised Ghanaian individuals living in rural and urban Ghana as well as in Amsterdam, the Netherlands; Berlin, Germany; and London, UK. Data collection for the study was standardized across all sites.^20^ Ethical approval of the study protocols was granted at all sites from the respective ethics committees in Ghana (School of Medical Sciences/Komfo Anokye Teaching Hospital Committee on Human Research, Publication & Ethical Review Board), the Netherlands (institutional review board of the Academic Medical Center, University of Amsterdam), Germany (ethics Committee of Charite-Universitätsmedizin Berlin), and the UK (London School of Hygiene and Tropical Medicine Research Ethics Committee) before data collection began in each country. There was no patient or public involvement in the design or analysis of this study. Written informed consent was obtained from each participant before enrollment; financial compensation was not provided. For the present analyses, only participants aged 25 to 75 years with complete data on SUA, microvascular, and macrovascular measurements (n = 4919) were included (Figure). eTable 1 in the Supplement reports the characteristics of the individuals included in (n = 4919) and excluded from (n = 792) the study. This study followed the Strengthening the Reporting of Observational Studies in Epidemiology (STROBE) reporting guideline.

**Figure.**
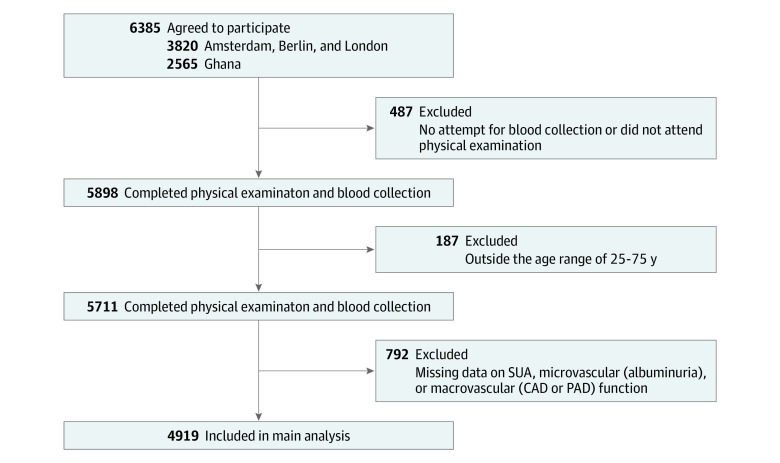
Flowchart of Study Design and Inclusion in Analyses CAD indicates coronary artery disease; PAD, peripheral artery disease; and SUA, serum uric acid.

### Assessments

The assessment of baseline sociodemographic and clinical characteristics and physical measurements, including the waist-hip ratio (WHR), body mass index (BMI), and blood pressure (BP), have been described in detail elsewhere.^10,20^ Hypertension was defined as systolic BP (SBP) greater than or equal to 140 mm Hg and/or diastolic BP (DBP) greater than or equal to 90 mm Hg and/or receiving antihypertensive medication. Ankle-brachial pressure index (ABI) measurements were performed in the supine position using a validated oscillometric device (WatchBP Office ABI; Microlife) with appropriately sized cuffs after at least 10 minutes of supine rest. Systolic BP was measured twice in the right and left brachial arteries and twice in the right and left posterior tibial arteries. Ankle-brachial pressure index was calculated by taking the highest arm SBP as the denominator and the lowest ankle BP as the numerator. The lowest of the left and right ABI measurements were used for analyses.

Biochemical measurements, including fasting glucose, hemoglobin (Hb) A_1c_, fasting lipids, and serum creatinine levels, have been described in detail elsewhere.^10,20^ Diabetes was defined according to the World Health Organization diagnostic criteria (self-reported diabetes, documented use of glucose-lowering medication, fasting plasma glucose levels ≥126 mg/dL [to convert to millimoles per liter, multiply by 0.0555], or HbA_1c_ ≥6.5% [to convert to proportion of total hemoglobin, multiply by 0.01]).^21^ The estimated glomerular filtration rate (eGFR) was calculated using the 2009 CKD Epidemiology Collaboration creatinine equation.^22^ The SUA concentration was measured using an enzymatic method (Trinder). The concentration of urinary albumin was measured by an immunochemical turbidimetric method (Roche Diagnostics), and urinary creatinine concentration was measured by a kinetic spectrophotometric method (Roche Diagnostics).

The diagnosis of macrovascular dysfunction was based on the presence of PAD or CAD. Peripheral artery disease was defined as ABI less than or equal to 0.90.^23^ In defining normal ABI measurement, an ABI greater than 1.4 was excluded because it could be suggestive of noncompressible vessels.^23^ Coronary artery disease was defined as self-reported myocardial infarction diagnosed by a physician and/or angina or myocardial infarction based on the Rose questionnaire.^24^ The Rose questionnaire has a high specificity to detect CAD and is valuable for screening individuals in large-scale epidemiologic surveys.^25^ The diagnosis of microvascular dysfunction was based on albuminuria, defined as albumin-creatinine ratio (ACR) greater than or equal to 3 mg/mmol (category≥A2) according to the 2012 Kidney Disease Improving Global Outcomes guideline.^26^

### Statistical Analysis

Participants were divided into quartiles of the measure of SUA (Q1, SUA ≤15.05 mg/dL [n = 1229]; Q2, SUA = 15.06-18.17 mg/dL [n = 1229]; Q3, SUA = 18.18-21.63 mg/dL [n = 1230]; and Q4, SUA ≥21.64 mg/dL [n = 1231]) (to convert SUA to micromoles per liter, multiply by 0.0595). Because of power limitations, we did not further stratify by the sites of residence. However, we used the site of residence as a covariate in our logistic regression models. Data with normal distributions are presented as mean (SD), whereas those not normally distributed are presented as median (IQR). Categorical data are presented as frequencies (percentages). For continuous variables, mean and median values were compared across SUA quartiles using 1-way analysis of variance for normally distributed variables and the Kruskal-Wallis test for variables not normally distributed. A χ^2^ test was used to compare categorical variables across SUA quartiles. Logistic regression analyses were used to examine the associations between elevated SUA concentrations (quartiles) and microvascular and macrovascular dysfunction, with adjustments for covariates. Participants in the first SUA quartile were defined as the reference group. Odds ratios (ORs) and their corresponding 95% CIs were estimated. In a sensitivity analysis, we assessed the associations between elevated SUA concentration based on the traditional cutoff values of hyperuricemia (SUA>7 mg/dL in men and >6 mg/dL in women).^6^ The minimal sufficient adjustment sets for estimating the direct effect size of SUA in microvascular and macrovascular dysfunction were determined by a directed acyclic graph.^27^ Based on the directed acyclic graph, 4 models were used to examine the data. Model 1 was unadjusted, model 2 was adjusted for age and sex, model 3 was additionally adjusted for eGFR, and model 4 was further adjusted for the site of residence, socioeconomic status, alcohol consumption, smoking, hypertension, diabetes, WHR, and total cholesterol concentration.

Mediation analysis was performed to assess the mediating roles of hypertension (SBP or DBP), hyperglycemia (HbA_1c_), systemic inflammation (hs-CRP concentration), or obesity (BMI or WHR) on the association between SUA concentrations and vascular dysfunction. Mediation analyses were performed only for vascular function outcomes that showed significant associations with SUA concentration. A statistical test of significance was set at a 2-tailed value of *P* < .05. Data were analyzed using SPSS, version 23 for Windows (IBM Corp). Mediation analysis was performed using the Hayes process, version 3.5 macro.^28^

## Results

### General Characteristics

Of the 4919 Ghanaian individuals included in the study, 3047 were women (61.9%) and 1872 were men (38.1%); mean (SD) age was 46.26 (11.08) years. Generally, higher SUA levels were associated with an increased risk of CVD (Table 1). For example, compared with the first quartile, levels in individuals in the fourth quartile were 14.0% higher for mean age (49.37 vs 43.29 years), 6.1% higher for WHR (0.93 vs 0.88), 10.6% higher for SBP (137.02 vs 123.94 mm Hg), 9.9% higher for DBP (85.47 vs 77.75 mm Hg), 7.1% higher for HbA_1c_ (5.8% vs 5.6%), and 6.7% higher for total cholesterol concentration (199.92 vs 187.16 mg/dL) (*P* < .001 for each). In line with these results, the rate of diabetes was 95% higher (17.5% vs 7.2%) and the rate of hypertension was 143% higher (62.1% vs 31.8%) in individuals in the fourth quartile compared with individuals in the first quartile (*P* < .001 for each). The median hs-CRP concentration in individuals in the fourth quartile was double that of individuals in the first quartile (0.10 vs 0.05 mg/dL; *P* < .001). The mean eGFR decreased across SUA quartiles, with eGFR varying by 15.32 mL/min/1.73 m^2^ between the first and fourth SUA quartiles (*P* < .001). There was a low proportion of individuals using urate-lowering medications (10 [0.2%]); the proportion of these individuals was similar in the SUA quartiles (eg, quartile 1, 2 [0.2%] vs quartile 4, 5 [0.4%]; *P* = .31).

**Table 1.** Baseline Characteristics of Study Participants by Serum Uric Acid Quartiles

Characteristic	No. (%)					*P* value for trend
	Overall	SUA, quartiles, mg/dL				
		Q1 (≤15.05)	Q2 (15.06-18.17)	Q3 (18.18-21.63)	Q4 (≥21.64)	
Participants	4919	1229	1229	1230	1231	NA
Age, mean (SD), y	46.26 (11.08)	43.29 (10.47)	45.29 (11.16)	47.06 (10.96)	49.37 (10.82)	<.001
Sex						
Women	3047 (61.9)	1112 (90.5)	911 (74.1)	640 (52.0)	384 (31.2)	<.001
Men	1872 (38.1)	117 (9.5)	318 (25.9)	590 (48.0)	847 (68.8)	
Higher educational level	507 (10.3)	83 (6.8)	107 (8.7)	141 (11.5)	176 (14.3)	<.001
Site of residence						
Ghana	2338 (47.5)	735 (59.8)	656 (53.4)	524 (42.6)	423 (34.4)	<.001
Europe	2581 (52.5)	494 (40.2)	573 (46.6)	706 (57.4)	808 (65.6)	
Urate-lowering medication	10 (0.2)	2 (0.2)	1 (0.1)	2 (0.2)	5 (0.4)	.31
Current smoking	146 (3.0)	13 (1.1)	23 (1.9)	44 (3.6)	66 (5.4)	<.001
Alcohol consumption, median (IQR), g/d	0.14 (0-2.02)	0.06 (0-0.88)	0.12 (0-1.57)	0.15 (0-2.28)	0.85 (0–6.18)	<.001
WHR, mean (SD)	0.90 (0.07)	0.88 (0.07)	0.89 (0.07)	0.90 (0.07)	0.93 (0.07)	<.001
BMI, mean (SD)	27.05 (5.45)	26.01 (5.17)	27.05 (5.49)	27.23 (5.51)	27.91 (5.45)	<.001
Obesity	1327 (27.0)	261 (21.2)	345 (28.1)	343 (27.9)	378 (30.7)	<.001
BP, mean (SD), mm Hg						
Systolic	130.09 (19.59)	123.94 (18.41)	127.75 (18.26)	131.64 (19.10)	137.02 (20.12)	<.001
Diastolic	81.40 (11.98)	77.75 (11.36)	80.09 (11.23)	82.29 (11.67)	85.47 (12.28)	<.001
Hypertension	2247 (45.7)	391 (31.8)	499 (40.6)	592 (48.1)	765 (62.1)	<.001
Diabetes	557 (11.3)	89 (7.2)	107 (8.7)	146 (11.9)	215 (17.5)	<.001
HbA_1c_, mean (SD), %	5.7 (3.3)	5.6 (3.4)	5.6 (3.3)	5.7 (3.2)	5.8 (3.2)	<.001
Lipids, mean (SD), mg/dL						
Total cholesterol	192.58 (43.70)	187.16 (40.60)	189.87 (41.38)	193.35 (44.08)	199.92 (47.95)	<.001
HDL-C	51.43 (13.92)	53.75 (13.53)	51.82 (13.92)	50.66 (13.53)	50.27 (14.31)	<.001
LDL-C	123.36 (37.90)	118.33 (34.42)	121.04 (35.19)	124.90 (37.90)	129.54 (42.92)	<.001
Triglycerides	89.46 (49. 60)	77.94 (38.97)	85.91 (47.83)	91.23 (51.37)	102.74 (56.68)	<.001
eGFR, mL/min/1.73 m^2^	95.14 (19.95)	102.45 (19.20)	96.81 (19.12)	94.18 (18.40)	87.13 (19.96)	<.001
hs-CRP, mg/dL	0.07 (0.02-0.25)	0.05 (0.01-0.20)	0.07 (0.02-0.23)	0.08 (0.02-0.26)	0.10 (0.03-0.32)	<.001

Abbreviations: BMI, body mass index (calculated as weight in kilograms divided by height in meters squared); BP, blood pressure; eGFR, estimated glomerular filtration rate; HbA_1c_, hemoglobin A_1c_; HDL-C, high-density lipoprotein cholesterol; hs-CRP, high sensitivity C-reactive protein; LDL-C, low-density lipoprotein cholesterol; NA, not applicable; Q, quartile; SUA, serum uric acid; WHR, waist-hip ratio.

SI unit conversion: To convert HbA_1c_ to proportion of total hemoglobin, multiply by 0.01; HDL-C, LDL-C, and total cholesterol to millimoles per liter, multiply by 0.0259; hs-CRP to milligrams per liter, multiply by 10; SUA to micromoles per liter, multiply by 0.0595; and triglycerides to millimoles per liter, multiply by 0.0113.

### SUA Level and Macrovascular/Microvascular Dysfunction

The unadjusted model showed a significant association between SUA quartiles and albuminuria (Table 2). The odds of albuminuria in the fourth SUA quartile were 56% higher than in individuals in the first SUA quartile (OR, 1.56; 95% CI, 1.19-2.04; *P* = .001). In the fully adjusted model, individuals in the fourth SUA quartile had higher odds of albuminuria compared with individuals in the first SUA quartile (adjusted OR [aOR], 1.54; 95% CI, 1.07-2.21; *P* = .02) but not peripheral artery disease (aOR, 1.35; 95% CI, 0.87-2.08) or CAD based on the Rose angina questionnaire (aOR, 1.09; 95% CI, 0.77-1.55). Among individuals in the fourth quartile, a higher z score SUA level was associated with higher odds of albuminuria in the unadjusted (aOR, 1.31; 95% CI, 1.03-1.67; *P* = .03) and age- and sex-adjusted (aOR, 1.35; 1.05-1.73; *P* = .02) models but not in the fully adjusted model (aOR, 0.99; 95% CI, 0.73-1.35; *P* = .95) (eTable 4 in the Supplement).

**Table 2.** Logistic Regression Models Among 4919 Individuals in the SUA Quartiles

Variable	OR (95% CI)			
	Model 1^a^	Model 2^b^	Model 3^c^	Model 4^d^
**Albuminuria**				
Q1 (≤15.05 mg/dL)	1 [Reference]	1 [Reference]	1 [Reference]	1 [Reference]
Q2 (15.06-18.17 mg/dL)	1.08 (0.81-1.44)	1.12 (0.83-1.49)	1.09 (0.81-1.46)	1.09 (0.79-1.51)
Q3 (18.18-21.63 mg/dL)	0.93 (0.69-1.26)	1.03 (0.76-1.40)	1.00 (0.73-1.36)	0.96 (0.68-1.36)
Q4 (≥21.64 mg/dL)	1.56 (1.19-2.04)	1.84 (1.36-2.49)	1.73 (1.26-2.37)	1.54 (1.07-2.21)
*P* value for trend	.001	<.001	<.001	.02
**PAD**				
Q1 (≤15.05 mg/dL)	1 [Reference]	1 [Reference]	1 [Reference]	1 [Reference]
Q2 (15.06-18.17 mg/dL)	0.92 (0.67-1.27)	1.00 (0.72-1.38)	0.98 (0.71-1.37)	1.07 (0.75-1.52)
Q3 (18.18-21.63 mg/dL)	0.86 (0.61-1.19)	1.04 (0.74-1.48)	1.02 (0.72-1.45)	1.20 (0.81-1.75)
Q4 (≥21.64 mg/dL)	0.85 (0.61-1.19)	1.19 (0.82-1.72)	1.14 (0.78-1.67)	1.35 (0.87-2.08)
*P* value for trend	.75	.78	.87	.57
**CAD**				
Q1 (≤15.05 mg/dL)	1 [Reference]	1 [Reference]	1 [Reference]	1 [Reference]
Q2 (15.06-18.17 mg/dL)	0.97 (0.75-1.26)	1.01 (0.77-1.31)	0.99 (0.76-1.29)	1.07 (0.81-1.41)
Q3 (18.18-21.63 mg/dL)	1.03 (0.79-1.33)	1.10 (0.84-1.45)	1.08 (0.82-1.42)	1.28 (0.95-1.73)
Q4 (≥21.64 mg/dL)	0.71 (0.54-0.94)	0.80 (0.59-1.09)	0.77 (0.56-1.06)	1.09 (0.77-1.55)
*P* value for trend	.04	.17	.13	.39

Abbreviations: CAD, coronary artery disease; OR, odds ratio; PAD, peripheral artery disease; Q, quarter; SUA, serum uric acid.

SI conversion: To convert SUA to micromoles per liter, multiply by 0.0595.

^a^ Unadjusted for any covariate.

^b^ Adjusted for age and sex.

^c^ Additionally adjusted for estimated glomerular filtration rate.

^d^ Further adjusted for the site of residence, socioeconomic status, alcohol consumption, smoking, diabetes, hypertension, waist-hip ratio, and total cholesterol level.

There was no significant association between SUA quartiles and PAD in the unadjusted (aOR, 0.85; 95% CI, 0.61-1.19; *P* = .75) and fully adjusted (aOR, 1.35; 95% CI, 0.87-2.08; *P* = .57) models. There was a significant association between increasing SUA quartiles and CAD in the unadjusted model; individuals in the fourth SUA quartile had lower odds of CAD (aOR, 0.71; 95% CI, 0.54-0.94; *P* = .04) compared with those in the first quartile. This association was no longer significant in the adjusted models, including the fully adjusted model (aOR, 1.09; 95% CI, 0.77-1.55; *P* = .39). In a sensitivity analysis using self-reported myocardial infarction alone, results were not significantly different in comparison with self-reported myocardial infarction and the Rose questionnaire (eTable 2 in the Supplement).

When we assessed the associations of elevated SUA concentration based on the traditional cutoff values (>7 mg/dL in men and >6 mg/dL in women) with albuminuria, PAD, and CAD, findings similar to the comparisons based on quartiles were observed (Table 3). In the fully adjusted model, elevated SUA level was significantly associated with albuminuria (aOR, 1.50; 95% CI, 1.14-1.98; *P* = .004) but not PAD (aOR, 1.35; 95% CI, 0.94-1.93; *P* = .10) or CAD (aOR, 1.02; 95% CI, 0.75-1.40; *P* = .88). Similar findings were obtained when SUA level was assessed as a continuous variable (Table 3). In the fully adjusted model, a 1-SD higher SUA concentration was associated with higher odds for albuminuria (aOR, 1.16; 95% CI, 1.02-1.32; *P* = .03) but not PAD (aOR, 1.09; 95% CI, 0.93-1.28; *P* = .29) or CAD (aOR, 1.06; 95% CI, 0.93-1.21; *P* = .36).

**Table 3.** Associations of SUA With Macrovascular and Kidney Microvascular Dysfunction

Variable	Model 1^a^		Model 2^b^		Model 3^c^		Model 4^d^	
	OR (95% CI)	*P* value	OR (95% CI)	*P* value	OR (95% CI)	*P* value	OR (95% CI)	*P* value
**Elevated SUA level** ^e^								
Albuminuria	1.81 (1.45-2.27)	<.001	1.77 (1.40-2.23)	<.001	1.70 (1.34-2.15)	<.001	1.50 (1.14-1.98)	.004
PAD	1.09 (0.81-1.48)	.56	1.17 (0.86-1.59)	.32	1.14 (0.83-1.56)	.42	1.35 (0.94-1.93)	.10
CAD	0.75 (0.57-0.97)	.03	0.78 (0.60-1.03)	.08	0.76 (0.58-1.00)	.05	1.02 (0.75-1.40)	.88
**SUA z score** ^f^								
Albuminuria	1.19 (1.08-1.31)	<.001	1.27 (1.14-1.41)	<.001	1.24 (1.11-1.38)	<.001	1.16 (1.02-1.32)	.03
PAD	0.91 (0.81-1.03)	.12	1.02 (0.89-1.17)	.78	1.00 (0.87-1.15)	.10	1.09 (0.93-1.28)	.29
CAD	0.89 (0.81-0.98)	.02	0.93 (0.83-1.04)	.18	0.91 (0.81-1.02)	.11	1.06 (0.93-1.21)	.36

Abbreviations: CAD, coronary artery disease; OR, odds ratio; PAD, peripheral artery disease; SUA, serum uric acid.

^a^ Unadjusted for any covariate.

^b^ Adjusted for age and sex.

^c^ Additionally adjusted for estimated glomerular filtration rate.

^d^ Further adjusted for the site of residence, socioeconomic status, alcohol consumption, smoking, diabetes, hypertension, waist-hip ratio, and total cholesterol level.

^e^ Association of elevated SUA levels with albuminuria, PAD, and CAD (reference is SUA level within the reference range). Elevated SUA level: greater than 7 mg/dL in men and greater than 6 mg/dL in women.

^f^ Association of SUA level *z* scores with albuminuria, PAD, and CAD.

### Mediation Analysis for SUA and ACR

Table 4 summarizes the effects of potential mediators on the association between SUA concentration and ACR z scores. In both the unadjusted and fully adjusted models, hs-CRP levels, WHR, and BMI did not mediate the association between SUA and ACR levels. The mediation effect size of HbA_1c_ observed in the unadjusted model was not found after full adjustment; a similar observation was made in a sensitivity analysis when individuals receiving glucose-lowering therapy were excluded (eTable 3 in the Supplement). However, SBP and DBP significantly mediated the association between high SUA concentrations and ACR z score in the unadjusted and fully adjusted models. In the fully adjusted model, SBP accounted for 19.4% and DBP accounted for 17.2% of the association between SUA concentrations and ACR z score. In a sensitivity analysis, the percentage of the effect size of BP was accentuated when individuals receiving antihypertensive therapy were excluded (eTable 3 in the Supplement).

**Table 4.** Potential Mediators of the Association Between SUA and ACR in 4919 Patients

Mediator	Effect size (95% CI)					
	Unadjusted model			Fully adjusted model^a^		
	Total effect of SUA on ACR	Indirect effect of SUA on ACR	% of Effect via mediator	Total effect of SUA on ACR	Indirect effect of SUA on ACR	% of Effect via mediator
SBP	0.00089 (0.00056 to 0.00122)	0.00039 (0.00022 to 0.00064)	43.8	0.00093 (0.00045 to 0.00141)	0.00018 (0.00007 to 0.00034)	19.4
DBP	0.00089 (0.00056 to 0.00122)	0.00039 (0.00020 to 0.00064)	43.8	0.00093 (0.00045 to 0.00141)	0.00016 (0.00006 to 0.00031)	17.2
WHR	0.00089 (0.00056 to 0.00122)	0.00001 (−0.00017 to 0.00015)	1.1	0.00078 (0.00031 to 0.00125)	−0.00003 (−0.00018 to 0.00008)	NA
BMI	0.00089 (0.00056 to 0.00122)	0.00001 (−0.00005 to 0.00006)	1.1	0.00078 (0.00031 to 0.00125)	−0.00008 (−0.00020 to 0.00003)	NA
HbA_1c_	0.00089 (0.00056 to 0.00122)	0.00008 (0.00003 to 0.00015)	9.0	0.00084 (0.00033 to 0.00136)	−0.00005 (−0.00012 to 0.00000)	NA
hs-CRP	0.00089 (0.00056 to 0.00122)	0.00001 (0.00000 to 0.00003)	1.1	0.00081 (0.00033 to 0.00129)	0.00003 (0.00000 to 0.00007)	3.8

Abbreviations: ACR, albumin-creatinine ratio; BMI, body mass index; DBP, diastolic blood pressure; HbA_1c_, hemoglobin A_1c_; hs-CRP, high-sensitivity C-reactive protein; NA, not applicable; SBP, systolic blood pressure; SUA, serum uric acid; WHR, waist-hip ratio.

^a^ Adjusted for age, sex, estimated glomerular filtration rate, site of residence, socioeconomic status, alcohol consumption, smoking, diabetes, hypertension, WHR, and total cholesterol level. Hypertension, diabetes, and WHR were excluded from the list of covariates when assessing the mediating roles of SBP or DBP, HbA_1c_, and obesity (BMI or WHR).

## Discussion

Using a representative sample of Ghanaian individuals, we noted that, among SSA individuals, higher SUA levels are associated with albuminuria independent of eGFR and a wide range of CVD risk factors. Increased SUA levels were not associated with higher odds of CAD or PAD. The association between elevated SUA concentrations and albuminuria was significantly mediated by SBP and DBP but not hs-CRP levels, HbA_1c_ levels, BMI, or WHR.

The association between SUA levels and albuminuria is in line with results reported in many European and Asian-origin populations,^5,6^ including Norwegian^29^ and Chinese^30^ individuals. Consistent with previous reports,^5,6^ our findings of an association between SUA levels and albuminuria in SSA individuals persisted after adjustment for a wide range of cardiometabolic risk factors. Although existing data have demonstrated a link between elevated SUA levels with albuminuria, it remains uncertain whether the outcome is causal. This uncertainty is evidenced by the lack of a clear mechanism by which SUA could cause microvascular injury to the kidney.^5,6,31^ Histologic evidence of urate crystal deposition in the kidney medullary interstitium in individuals with hyperuricemia suggests causality.^31^ However, the characteristic kidney findings in urate deposition, including advanced arteriolosclerosis and glomerulosclerosis, are indistinguishable from those observed in other conditions, including long-standing hypertension and advancing age.^6^ In the present study, the persistence of the association between elevated SUA levels and albuminuria after adjustment for a wide range of CVD risk factors seems to suggest that an association between elevated SUA levels and kidney microvascular dysfunction cannot be precluded. However, attenuation in the strength of the association after adjusting for CVD risk factors suggests that the association between SUA and kidney microvascular dysfunction could be partly mediated by CVD risk factors.

Consistent with the hyperuricemic hypertension hypothesis,^6^ our findings support the idea that elevated SUA levels causing kidney microvascular dysfunction could occur via a mechanism partly linked to elevated BP. High extracellular SUA levels may promote intracellular SUA invasion via specific transporters, which may, in turn, increase the risk of vascular disease.^7^ Extracellular urate may be deposited in the vasculature, where it forms a nidus for vascular calcification, increasing the risk of hypertension—a key risk factor for albuminuria.^7^ In an experimental model, a mild increase in SUA concentration was associated with hypertension and kidney injury^32^; this finding was supported by a clinical study.^33^ There is, however, conflicting experimental evidence that elevated SUA concentration in the setting of normotension is still associated with kidney microvascular dysfunction.^34^ Therefore, elevated SUA levels could injure the kidneys via mechanisms not related to hypertension. Mechanistically, high SUA concentrations may predispose to increased SUA urinary excretion, which could lead to kidney injury, especially in the setting of dehydration or heat stress.^7^

It remains uncertain whether injury to the kidney microvasculature from elevated SUA levels, whether directly or mediated through another variable, is reversible or improves with lowering of SUA levels. Although a meta-analysis by Chen et al^35^ failed to note sufficient evidence of improvement in kidney function outcomes following urate-lowering therapy, urate-lowering therapy attenuated the decrease in the slope of eGFR. Based on the fact that most studies assessing the role of SUA level–lowering therapy have focused on individuals with an existing or high risk of kidney disease,^35,36,37^ it may be valuable to assess the role in individuals with preserved kidney function. Experimental data show that SUA-induced hypertension is fully reversible by uric acid–lowering agents if kidney function is preserved; with the onset of kidney disease, hypertension becomes salt sensitive and SUA independent.^7^

Excluding individuals receiving antihypertensive therapy from the analysis increased the mediating effect size of BP in the association between SUA levels and albuminuria. Although the importance of this finding is not clear, it could suggest that pharmacologic control of BP obscured the association between elevated SUA levels and albuminuria. Longitudinal studies are needed to better characterize antihypertensive therapy in terms of the association between elevated SUA levels and albuminuria.

Our observation that HbA_1c_ and hs-CRP concentrations did not significantly mediate the association between SUA concentration and albuminuria contrasts with experimental data showing that elevated SUA concentration may induce a chronic inflammatory response, potentially leading to kidney microvascular endothelial injury.^38^ Although some authors have reported that elevated SUA levels independently suggest the probable development of microvascular complications in the setting of diabetes, there is no consensus on the role of SUA in glycemic control.^6^ Waist-hip ratio and BMI did not significantly mediate the association between the SUA level and ACR z score. A small sample-sized, population-based cohort study reported that SUA levels are inversely associated with adiponectin, a fat-derived hormone protective against cardiometabolic disease.^18^ Based on this previous finding and the role of central obesity as an independent risk factor for albuminuria,^39^ it remains unclear why WHR and BMI did not mediate the association between SUA levels and ACR. Further studies assessing the mediating roles of other measures of obesity aside from WHR and BMI could be valuable.

Although statistically nonsignificant, after full adjustment, our findings on elevated SUA levels and CAD agree with a large, multiethnic, population-based study in the US that concluded that elevated SUA levels were not independently associated with CAD.^40^ Some authors argue that any apparent association between SUA levels and CAD is probably due to the association of SUA level with other risk factors or the inability to sufficiently adjust for vascular risk factors.^6^ This position is challenged by findings from other studies,^5,6^ including those among Chinese^41^ and Dutch^15^ individuals, in which increased SUA levels were independently associated with CAD. The epidemiologic evidence linking elevated SUA levels with CAD thus remains uncertain.

Our observed lack of association between elevated SUA concentration and PAD is consistent with a previous report in a multiethnic cohort in the US without diabetes that showed that a history of gout, but not elevated SUA levels, was significantly associated with PAD.^42^ However, our findings contrast with other studies, including those among Taiwanese individuals,^43^ Scottish individuals,^44^ and a US general population based on the National Health and Nutrition Examination Survey data^45^ in which elevated SUA levels were a significant and independent predictor of PAD. Although racial and ethnic differences cannot be ruled out, disparities among prior findings could reflect a misclassification of PAD based on the recommended diagnostic criteria (ABI ≤0.9).^23^ The few studies evaluating the association between SUA levels and PAD have differed in the proportion of individuals with diabetes included.^42,43^ In diabetes, hardening or noncompressibility of the distal arteries could lead to an increase of ABI.^23^ Therefore, individuals with PAD with noncompressible distal arteries may have ABI greater than 0.9, thereby masking PAD diagnosis based on ABI measurement.

In this study, we used a sample of Ghanaian individuals to represent the SSA population. To enhance the generalizability of our study findings, it is important to replicate this study in other SSA populations, especially those living in Eastern and Southern Africa. In addition to environmental differences, SSA populations are known to exhibit substantial genetic diversity.^46^

### Limitations

The study has limitations. First, the cross-sectional design limits us from excluding reverse causation. Mechanistically, kidney microvascular dysfunction may alter extracellular fluid volume as well as proximal tubular urate absorption; both factors could result in increased SUA levels.^47^ Second, there are limitations in performing mediation analyses in cross-sectional data (and binary outcomes) owing to the absence of temporality. Implicitly underlying all mediation methods is a temporal component.^48,49^ To overcome limitations associated with cross-sectional data and binary outcome variables, we used the product of coefficients approach as recommended for this situation.^50^ Longitudinal studies are warranted to examine the temporal order of the association between SUA levels and albuminuria and potential mediators in this association. Third, advanced imaging modalities were not performed in the evaluation of CAD or PAD owing to feasibility. Fourth, other microvascular diseases, including retinopathy and neuropathy, were not assessed in this study.

## Conclusions

Our study suggests that higher SUA concentration is associated with kidney microvascular dysfunction but not macrovascular dysfunction in SSA individuals. This association between SUA concentration and kidney microvascular dysfunction is mediated partly through BP. Our findings suggest that SSA individuals with elevated SUA levels may benefit from periodic screening for kidney microvascular dysfunction to aid early detection and treatment. Our study also provides mechanistic insights into vascular dysfunction in SSA individuals and opportunities for research aimed at vascular disease risk prevention and/or treatment.

## Notes

Supplement. **eTable 1.** Comparison of Baseline Characteristics of Individuals Included and Excluded From the Current Analysis **eTable 2.** Logistic Regression Models for CAD (Based on a Previous History of MI or Use of the Rose Angina Questionnaire Alone) Among Individuals in the SUA Quartiles **eTable 3.** Mediation Effects of SBP, DBP, and HbA_1c_ Concentration on the Association Between SUA Concentration and ACR z Score for Individuals Not on Antihypertensive Therapy (n = 3894) or Hypoglycemic Medication (n = 4693) **eTable 4.** Associations of z Score SUA With Albuminuria, PAD, and CAD in Individuals in the Fourth SUA Quartile
