# Supplementary material for: Associations of Serum Uric Acid Levels With Macrovascular and Renal Microvascular Dysfunction Among Individuals From Sub-Saharan Africa
Source: JAMA Netw Open. 2021 Oct 14;4(10):e2128985. doi: 10.1001/jamanetworkopen.2021.28985 (PMC8517747; doi:10.1001/jamanetworkopen.2021.28985)
Supplement: Supplement. — eTable 1. Comparison of Baseline Characteristics of Individuals Included and Excluded From the Current Analysis eTable 2. Logistic Regression Models for CAD (Based on a Previous History of MI or Use of the Rose Angina Questionnaire Alone) Among Individuals in the SUA Quartiles eTable 3. Mediation Effects of SBP, DBP, and HbA1c Concentration on the Association Between SUA Concentration and ACR z Score for Individuals Not on Antihypertensive Therapy (n = 3894) or Hypoglycemic Medication (n = 4693) eTable 4. Associations of z Score SUA With Albuminuria, PAD, and CAD in Individuals in the Fourth SUA Quartile [file jamanetwopen-e2128985-s001.pdf]

**Supplementary Online Content**

Hayfron-Benjamin CF, van den Born BJ, Amoah AGB, et al. Associations of serum uric acid levels with macrovascular and renal microvascular dysfunction among individuals from Sub-Saharan Africa. *JAMA Netw Open*. 2021;4(10):e2128985. doi:10.1001/jamanetworkopen.2021.28985

**eTable 1.** Comparison of Baseline Characteristics of Individuals Included and Excluded From the Current Analysis

**eTable 2.** Logistic Regression Models for CAD (Based on a Previous History of MI or Use of the Rose Angina Questionnaire Alone) Among Individuals in the SUA Quartiles

**eTable 3.** Mediation Effects of SBP, DBP, and $HbA_{1c}$ Concentration on the Association Between SUA Concentration and ACR z Score for Individuals Not on Antihypertensive Therapy (n = 3894) or Hypoglycemic Medication (n = 4693)

**eTable 4.** Associations of z Score SUA With Albuminuria, PAD, and CAD in Individuals in the Fourth SUA Quartile

This supplementary material has been provided by the authors to give readers additional information about their work.

© 2021 Hayfron-Benjamin CF et al. *JAMA Network Open.*

eTable 1: Comparison of baseline characteristics of individuals included and excluded from the current analysis†.

| | Included | Excluded | p-value |
|---|---|---|---|
| | *4919* | 792 | |
| Age, y | 46.26 (±11.08) | 47.81 (±11.12) | <0.001 |
| Female sex (%) | | | 0.502 |
| Male | 1872 (38.1%) | 291 (36.7%) | |
| Female | 3047 (61.9%) | 501 (63.3%) | |
| Higher education (%) | 507 (10.3%) | 72 (9.1%) | 0.311 |
| Site of residence (%) | | | <0.001 |
| Ghana | 2338 (47.5%) | 194 (24.5%) | |
| Europe | 2581(52.5%) | 598 (75.5%) | |
| Current smokers (%) | 146 (3.0%) | 12 (2.9%) | 0.959 |
| Alcohol intake, g/day* | 0.14 (2.02) | 0.12 (1.83) | 0.847 |
| WHR | 0.90 (±0.07) | 0.90 (±0.09) | 0.116 |
| BMI, kg/m$^2$ | 27.05 (±5.45) | 28.20 (±5.46) | <0.001 |
| Systolic BP, mmHg | 130.09 (±19.59) | 134.70 (±19.21) | <0.001 |
| Diastolic BP, mmHg | 81.40 (±11.98) | 83.11 (±11.56) | <0.001 |
| Hypertension (%) | 2247 (45.7%) | 421 (53.2%) | <0.001 |
| Diabetes (%) | 557 (11.3%) | 71 (9.0%) | 0.050 |
| $HbA_{1c}$, mmol/mol | 38.34 (±12.32) | 39.23 (±11.06) | 0.072 |
| Total cholesterol, mmol/l | 4.98 (±1.13) | 4.93 (±1.10) | 0.283 |
| Triglycerides, mmol/l | 1.01 (±0.56) | 0.97 (±0.47) | 0.045 |
| HDL-cholesterol, mmol/l | 1.33 (±0.36) | 1.32 (±0.35) | 0.266 |
| LDL-cholesterol, mmol/l | 3.19 (±0.98) | 3.18 (±0.94) | 0.750 |
| eGFR, ml/min/1.73m$^2$ | 95.14 (±19.95) | 92.29 (±19.98) | 0.001 |
| hs-CRP, mg/L * | 0.70 (2.30) | 0.80 (2.63) | 0.754 |

Data are mean (± standard deviation), median (interquartile range), or n (%).
Abbreviations: BMI = body mass index; BP = blood pressure, HbA1c = glycated hemoglobin; HDL = high density lipoprotein; hs-CRP = high sensitivity C-reactive protein, LDL = low density lipoprotein.
*Data presented as median (interquartile range).

†Individuals excluded from the current analyses were aged 25-75 years but had incomplete data on serum uric acid, microvascular, and macrovascular measurements.

© 2021 Hayfron-Benjamin CF et al. *JAMA Network Open.*

eTable 2: Logistic regression models for CAD (based on a previous history of MI or use of the Rose Angina questionnaire alone) among individuals in the SUA quartiles (reference is SUA Q1)

| | OR (95% CI), p-value | | | |
|---|---|---|---|---|
| | Model 1 | Model 2 | Model 3 | Model 4 |
| CAD based on a history of MI diagnosed by a doctor (n=4872) | | | | |
| Q1 ((≤ 253.0 μmol/L) | 1.00 (Reference) | 1.00 (Reference) | 1.00 (Reference) | 1.00 (Reference) |
| Q2 (253.1-305.3 μmol/L) | 0.78 (0.58-1.03) | 0.81 (0.60-1.08) | 0.80 (0.60-1.07) | 0.88 (0.65-1.19) |
| Q3 (305-4-363.6 μmol/L) | 0.67 (0.50-0.90) | 0.75 (0.55-1.02) | 0.74 (0.54-1.01) | 0.98 (0.70-1.37) |
| Q4 (≥ 363.7 μmol/L) | 0.61 (0.45-0.83) | 0.74 (0.52-1.04) | 0.71 (0.50-1.01) | 1.11 (0.76-1.64) |
| *p-value for trend* | *0.007* | *0.204* | *0.167* | *0.634* |
| | | | | |
| CAD based on ROSE angina questionnaire (n = 4919) | | | | |
| | | | | |
| Q1 ((≤ 253.0 μmol/L) | 1.00 (Reference) | 1.00 (Reference) | 1.00 (Reference) | 1.00 (Reference) |
| Q2 (253.1-305.3 μmol/L) | 0.97 (0.75-1.26) | 1.01 (0.77-1.31) | 0.99 (0.76-1.29) | 1.07 (0.81-1.41) |
| Q3 (305-4-363.6 μmol/L) | 1.03 (0.79-1.33) | 1.10 (0.84-1.45) | 1.08 (0.82-1.42) | 1.28 (0.95-1.73) |
| Q4 (≥ 363.7 μmol/L) | 0.71 (0.54-0.94) | 0.80 (0.59-1.09) | 0.77 (0.56-1.06) | 1.09 (0.77-1.55) |
| *p-value for trend* | *0.043* | *0.166* | *0.131* | *0.393* |

Abbreviations: CAD = coronary artery disease; MI = myocardial infarction; SUA = serum uric acid.

© 2021 Hayfron-Benjamin CF et al. *JAMA Network Open.*

eTable 3: Mediation effects of SBP, DBP, and HbA1c concentration on the association between SUA concentration and ACR Z-score for individuals not on antihypertensive therapy (n = 3894) or hypoglycemic medication (n=4693)

| Mediator | Unadjusted Model | | | Fully Adjusted Model* | | |
|---|---|---|---|---|---|---|
| | Total effect of SUA on ACR | Indirect effect(s) of SUA on ACR | % of effect via mediator | Total effect of SUA on ACR | Indirect effect(s) of SUA on ACR | % of effect via mediator |
| SBP | 0.00037 (0.00001, 0.00073) | 0.00036 (0.00014, 0.00067) | 97.3% | 0.00044 (-0.00010, 0.00098) | 0.00017 (0.00004, 0.00036) | 38.6% |
| DBP | 0.00037 (0.00001, 0.00073) | 0.00038 (0.00014, 0.00073) | 102.7% | 0.00044 (-0.00010, 0.00098) | 0.00017 (0.00004, 0.00036) | 38.6% |
| HBA1c | 0.00078 (0.00043, 0.00113) | 0.00005 (0.00000, 0.00011) | 6.4% | 0.00085 (0.00034, 0.00137) | -0.00003 (-0.00008, 0.00000) | -3.5% |

Fully adjusted model: adjusted for age, sex, eGFR, site of residence, socioeconomic status, alcohol intake, smoking, diabetes, hypertension, WHR, and total cholesterol,

*Hypertension and diabetes were respectively excluded from the list of covariates when assessing the mediating roles of blood pressure (SBP or DBP) and HbA1c.

Abbreviations: ACR = albumin-creatinine ratio; DBP = diastolic blood pressure; HbA1c = glycated hemoglobin; SUA = serum uric acid; SBP = systolic blood pressure; WHR = waist to hip ratio.

© 2021 Hayfron-Benjamin CF et al. *JAMA Network Open.*

eTable 4: Associations of Z-score SUA with albuminuria, PAD, and CAD in individuals in the fourth SUA quartile

| | OR (95% CI), p-value | | | |
|---|---|---|---|---|
| | Model 1 | Model 2 | Model 3 | Model 4 |
| Albuminuria | 1.31 (1.03-1.67), 0.030 | 1.35 (1.05-1.73), 0.019 | 1.12 (0.86-1.46), 0.407 | 0.99 (0.73-1.35), 0.954 |
| PAD | 0.83 (0.55-1.24), 0.366 | 0.86 (0.57-1.29), 0.466 | 0.89 (0.58-1.35), 0.573 | 1.01 (0.64-1.60), 0.965 |
| CAD | 0.80 (0.56-1.13), 0.206 | 0.82 (0.57-1.17), 0.269 | 0.74 (0.51-1.08), 0.120 | 0.89 (0.60-1.32), 0.573 |

Model 1, unadjusted for any covariate; model 2, adjusted for age and sex, model 3, additionally adjusted for eGFR; and model 4, further adjusted for the site of residence, socioeconomic status, alcohol consumption, smoking, diabetes, hypertension, waist-to-hip ratio, and total-cholesterol concentration,

Abbreviations: eGFR = estimated glomerular filtration rate; CAD = coronary artery disease; PAD = peripheral artery disease; SUA = serum uric acid.

© 2021 Hayfron-Benjamin CF et al. *JAMA Network Open.*
